# Multi-frequency impedance sensing for detection and sizing of DNA fragments

**DOI:** 10.1038/s41598-021-85755-9

**Published:** 2021-03-22

**Authors:** Jianye Sui, Neeru Gandotra, Pengfei Xie, Zhongtian Lin, Curt Scharfe, Mehdi Javanmard

**Affiliations:** 1 Department of Electrical and Computer Engineering, Rutgers University, New Brunswick, 94 Brett Rd, Piscataway, NJ 08854 USA; 2 Department of Genetics, Yale University School of Medicine, 333 Cedar Street, PO Box 208005, New Haven, CT 06520-8005 USA

**Keywords:** Nanobiotechnology, Biosensors

## Abstract

Electronic biosensors for DNA detection typically utilize immobilized oligonucleotide probes on a signal transducer, which outputs an electronic signal when target molecules bind to probes. However, limitation in probe selectivity and variable levels of non-target material in complex biological samples can lead to nonspecific binding and reduced sensitivity. Here we introduce the integration of 2.8 μm paramagnetic beads with DNA fragments. We apply a custom-made microfluidic chip to detect DNA molecules bound to beads by measuring Impedance Peak Response (IPR) at multiple frequencies. Technical and analytical performance was evaluated using beads containing purified Polymerase Chain Reaction (PCR) products of different lengths (157, 300, 613 bp) with DNA concentration ranging from 0.039 amol to 7.8 fmol. Multi-frequency IPR correlated positively with DNA amounts and was used to calculate a DNA quantification score. The minimum DNA amount of a 300 bp fragment coupled on beads that could be robustly detected was 0.0039 fmol (1.54 fg or 4750 copies/bead). Additionally, our approach allowed distinguishing beads with similar molar concentration DNA fragments of different lengths. Using this impedance sensor, purified PCR products could be analyzed within ten minutes to determine DNA fragment length and quantity based on comparison to a known DNA standard.

## Introduction

Miniaturized technologies for quantification and sizing of nucleic acids can serve as point-of-use tools for research and clinical applications ranging from infectious and genetic disease testing to screening of environmental samples for viral and pathogen detection^1,2^. Gold standard techniques for highly sensitive DNA quantification are optical in nature and generally require bulky instrumentation for readout, such as fluorescence^3–5^ and plasmonic based detection^6–8^. Optical imaging technologies have been miniaturized to detect fluorophores for on-chip optofluidic microscopy^9,10^, portable tomographic microscopy^11^ and cell-phone based microscopy^12,13^. More recently, the use of deep-learning in conjunction with cell-phone microscopy has enabled development of portable tools for analyzing nucleic acids^14^. Though much progress has been made in building portable optical systems, ultra-compact portable^15–18^ and wearable^19,20^ devices can be more readily achieved by using electrical biosensors due to the comparative ease in miniaturizing electronic systems.

Electrochemical quantification of DNA typically relies on the addition of a redox probe molecule^21,22^ or an enzymatic reaction^23^ as implemented using screen-printed electrodes, carbon nanotubes, conductive polymers, and gold nanoparticles on complementary metal–oxide–semiconductor (CMOS) substrates^24–27^. Prior work with graphene-based electrodes for nucleic acid quantification has relied on the electron transfer properties of the nucleic acids themselves^28,29^. Electronic flow-through measurements (e.g., impedance cytometry, nanopores) detect and quantify analytes (cells, particles, molecules) passing through a micro- or nanoscale aperture based on real-time monitoring of direct changes in electrical properties such as impedance, resistance/conductance, charge, or other dielectric properties^30–34^. Recently, tunable resistive pulse sensing was used to measure surface potential of paramagnetic beads coated with DNA at DC^35^. A variety of nanopore based geometries have been utilized for quantifying and sizing of nucleic acids strands based on measurable differences in electric current during DNA translocation across nanopores^36,37^. While highly sensitive, the precise manufacturing of nanoscale pores with high-yield and robust operation resilient to clogging and device failure still remains a challenge^38^. Instead of using nanopores, Esfandiari et al. achieved sequence-specific DNA detection by investigating the blocked current of the bead hybridizing with a target DNA^39^. Previously, Sohn et al. reported the use of resistive pulse sensing across microscale pores for protein detection^40^, where quantification relied on the use of target antigen binding to antibody coated colloids, and detecting changes in DC current across pores as a result of binding. However, the use of this technique for consistent and repeatable detection and quantification of nucleic acid strands with DC or low-frequency (< 100 kHz) is challenging, particularly for shorter DNA strands (< 100 bp).

Here, we introduce a novel approach to identify DNA fragments based on their frequency-dependent dielectric properties. We use multi-frequency excitation of electric fields within a small microfluidic PDMS channel to detect changes in electrical impedance as DNA fragments, which are coupled on micron-sized particles, pass through the channel. By combining PCR-based amplification with electrical detection, non-target sequences are removed prior to detection in order to maximize the sensitivity and specificity for DNA target analysis. To investigate the ability of this technology to distinguish DNA fragments of different quantity and size at high sensitivity, we studied the effect of different DNA concentrations and fragment lengths on frequency-dependent electrical impedance changes. We show that this electronic biosensor could be used to rapidly detect and size DNA fragments at the femtomole level over a 100-fold dynamic range. After further development, we envision a future application of this technology for detection of PCR amplification products from ultra-low DNA input amounts (e.g., newborn dried blood spots, infectious diseases samples) that may not be detectable using gel-electrophoresis and thus require additional instrumentation for readout (e.g., qPCR, next-generation sequencing).

## Results

### Device design

**Figure 1:**
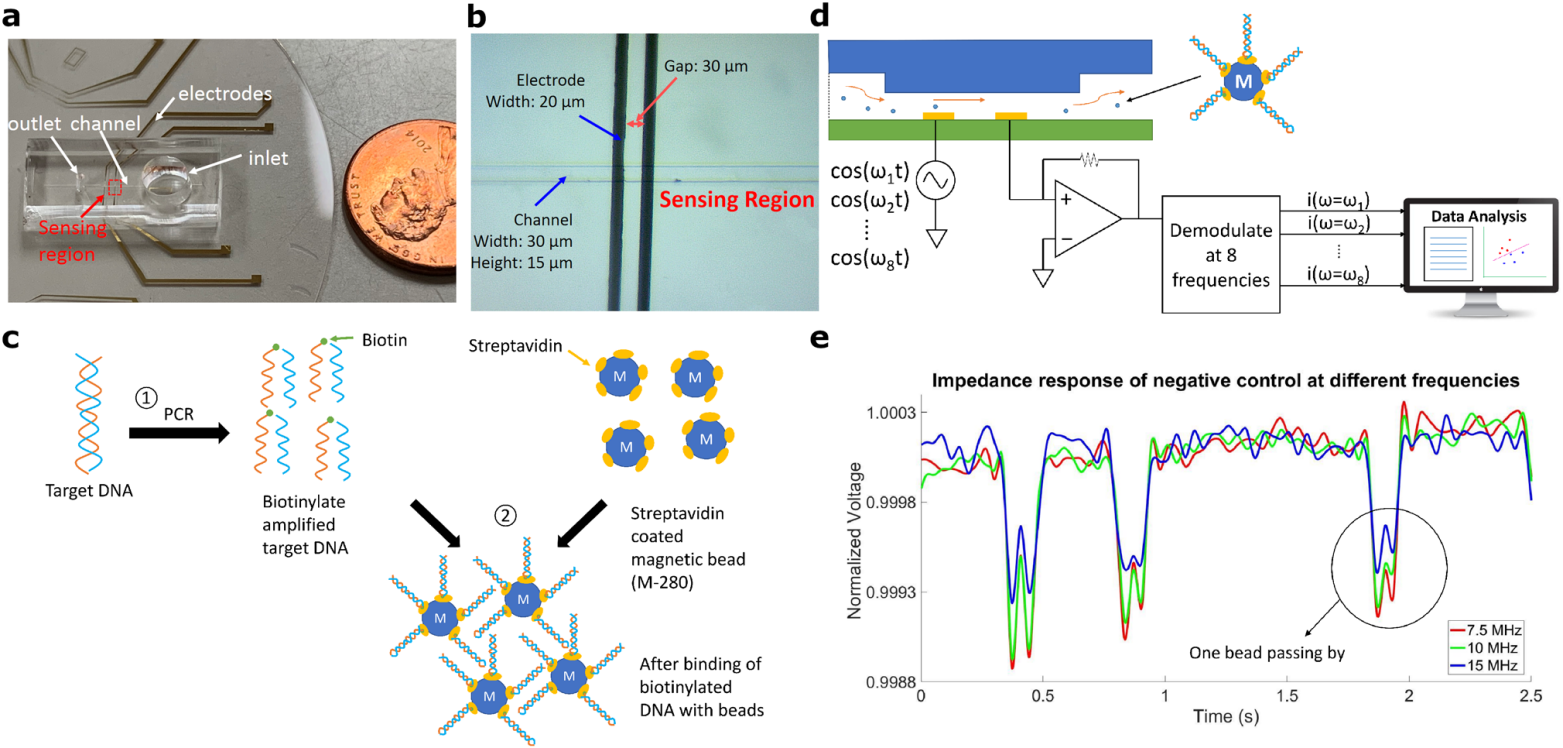
Strategy overview. (**a**) Image of the device in which a soft-lithography made PDMS is integrated with the electrode patterned on the fused silicon wafer. (**b**) The microscope image of channel and electrodes. (**c**) The sample preparation includes ① Amplifying target DNA using polymerase chain reaction (PCR). ② Immobilizing biotinylated target DNA with streptavidin coated paramagnetic beads. ③ Washing and resuspending. (**d**) The schematic diagram of detection. The bead is injected from inlet well using micro pipette. As beads flow through the pore, the impedance change is captured by the lock-in amplifier at multiple frequencies. The data are sent to the PC and analyzed in Matlab. (**e**) Representative data of bare paramagnetic beads passing through the sensing region measured at 7.5 MHz, 10 MHz, and 15 MHz respectively.

Our device is designed to perform multi-frequency impedance cytometry for direct detection of DNA captured on paramagnetic beads. The basic device consists of two layers covalently bonded together. The first layer is a microfluidic channel made of polydimethylsiloxane (PDMS), and the second layer is a pair of electron beam-deposited reusable coplanar gold electrodes on a fused silica substrate (Fig. 1a,b). Each electrode is 20 μm wide and the gap between the two electrodes is 30 μm. The sensitivity of the detector increases as the diameter of the channel approaches the size of the bead, while in turn the risk of clogging significantly increases as the channel becomes too small. The micro-channel was designed with the dimension of 30 μm in width and 15 μm in height, which is large enough to minimize clogging and small enough to obtain sufficient sensitivity during measurements.

### Electrical impedance sensing

We performed multi-frequency impedance cytometry to detect the impedance difference of beads coupled with different DNA amounts. When a bead flows through the sensing region, it partially impedes the AC electric field generated between the two electrodes, which results in an instantaneous frequency-dependent drop in ionic current, i.e., a momentary increase in impedance. Using a multi-frequency lock-in amplifier (Zurich Instruments HF2A, Zurich, Switzerland), the impedance response was measured simultaneously at 8 different frequencies ranging from 100 kHz to 20 MHz. One electrode was excited with a combination of 8 different frequency AC signals and the other electrode was tied to a transimpedance amplifier (Fig. 1d). Different quantities and sizes of DNA immobilized onto paramagnetic beads not only modulate the overall diameter of the DNA beads as compared to bare beads, but also influence surface potential, conductance, and permittivity. Each of these physical parameters was associated with differences in impedance measurements at different frequencies. Figure 1e shows the representative multi-frequency (7.5 MHz, 10 MHz and 15 MHz) time series data of bare paramagnetic beads in a 2.5 s time window. Traces are normalized with respect to the baseline impedance for ease of comparison. Each double peak observed corresponds to a single bead passing over the two electrodes. The reason for the double peak is due to the fact that when a bead flows directly above one electrode, a relative larger perturbation to the electric field is created compared to the bead flowing through the space between the two electrodes, thus resulting in two smaller peaks superimposed on a large peak. The bead can be modeled as a capacitor and a resistor in series with each other, which is parallel to the resistance of the solution. As frequency increases greater than 5 MHz, the impedance decreases due to the parasitic capacitance, which creates a path for the electric field parallel to the conductive solution, thus making the beads more permeable to the electric field generated by the electrodes. The impedance difference between beads containing DNA compared to bare beads without DNA was significantly larger at higher frequencies (> 500 kHz), due to the dominance of surface potential, conductance, and permittivity in impedance, as opposed to lower frequencies where impedance peak response (IPR) was primarily dictated by bead diameter. The summing of peak intensities of each bead across the frequency spectrum enabled maximum differentiation between different bead populations. To determine the physical mechanism that allows for bead differentiation in the frequency region of interest, we performed measurements on beads of the same size but with differing surface potentials (See SI Appendix, Fig. S1). Beads with larger surface potential displayed a higher average distribution of IPR, suggesting that one of the mechanisms for DNA detection was a change in surface potential. Previous work^40^ involving DC current measurements relied on changes in diameter. We have tested 3 different types of magnetic beads (M270, M280 and C1). Based on the properties and the nature of our sensor we chose to proceed with M280.

### DNA detection by impedance cytometry

#### Testing of 300 bp DNA beads

**Figure 2:**
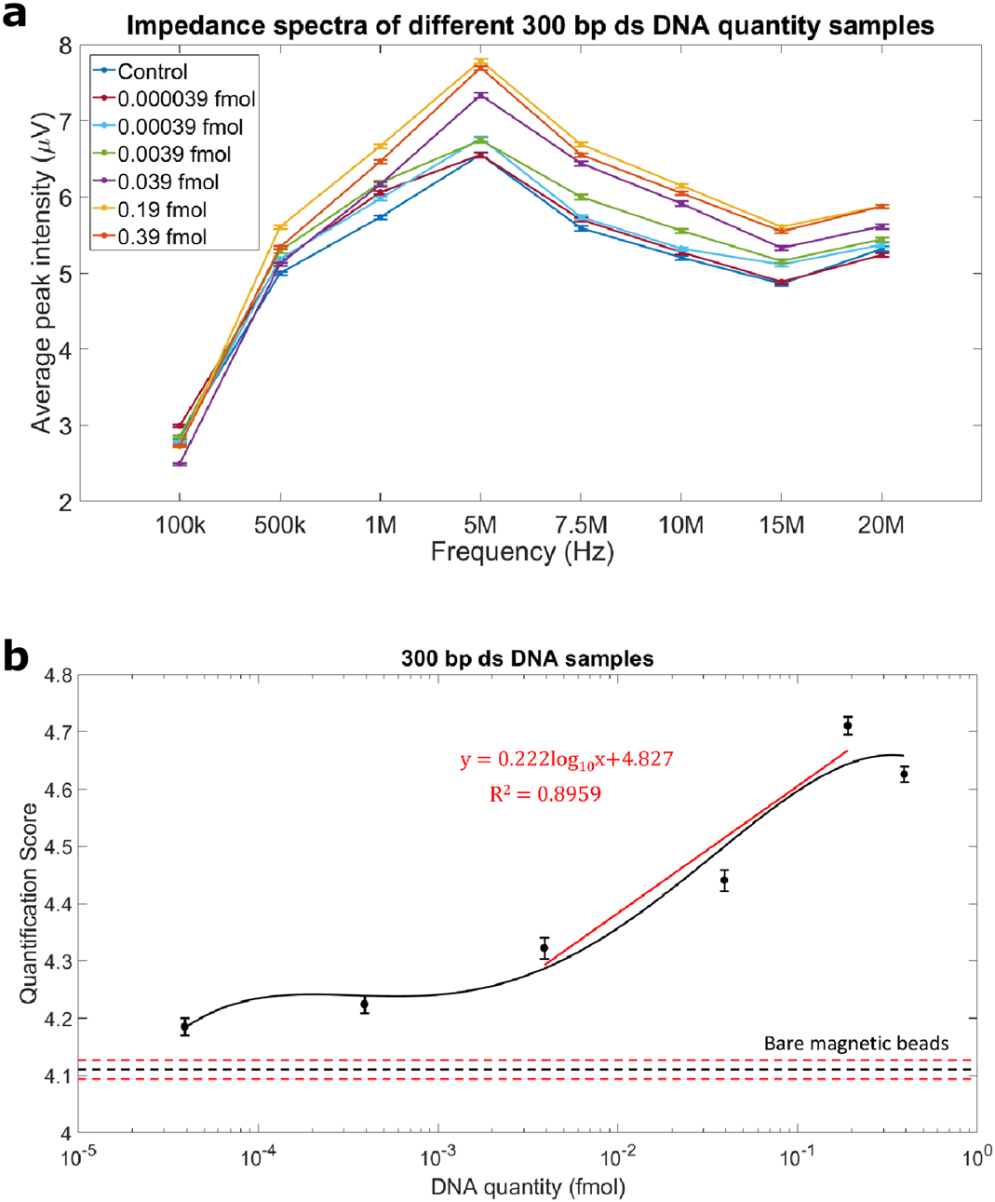
Effect of DNA concentration attached to the bead on impedance response. (**a**) Impedance spectra for beads attached with different concentrations of 300 bp ds DNA (Table 1) and negative control beads (paramagnetic M280, Invitrogen). For all samples measured, impedance peak response increased as frequency increased from 100 kHz to 5 MHz and decreased as frequency increased to 15 MHz. (**b**) The quantification score of different 300 bp DNA beads and negative control was calculated based on impedance responses measured at 8 frequencies. The black solid line shows the polynomial fitted curve across the 6? different DNA concentrations. The red line shows the linear correlations between DNA quantity and quantification score in the linear region. Error bars represent standard error of mean. Horizontal black and red dotted lines represent the average quantification score of the negative control samples and negative control ± standard error, respectively.

Measurements of beads with different DNA concentrations were performed to study the effect of different DNA amounts on frequency-dependent impedance sensing. A stock solution of beads coated with 300 bp DNA fragments was prepared of which only a small sample amount was used for impedance cytometry (Table 1). The number of DNA molecules per bead is an average estimation across approximately 500 beads. This number was obtained according to our previous works^20,41^. The current design of the device requires a minimum volume to maintain free flow in the PDMS microfluidics channel. In order to test the sensitivity of the device for detecting small DNA amounts, a one microliter aliquot of the DNA coated beads was diluted in 60 μL of phosphate buffered saline (PBS). Due to its relatively high salt concentration and increased conductivity, PBS has been shown to increase the sensitivity in impedance measurements^42^. Figure 2a shows the impedance spectra of beads with different DNA amounts measured at 8 frequencies. For each of the DNA concentrations measured, the average IPR gradually increased in value from 100 kHz to 5 MHz and decreased in value from 7 to 15 MHz. There was a positive correlation between DNA amounts and average IPR. At higher frequencies, the IPR of the negative control (bare streptavidin-coated paramagnetic beads) overlapped with that of beads with low DNA amounts. These findings showed the positive correlation of DNA amounts attached to beads with IPR. We observed a linear increase in average IPR for DNA concentrations up to 0.19 fmol. Increased DNA amounts resulted in a higher surface potential of the beads, which was associated with a larger impedance difference compared to control bare bead.

**Table 1:** DNA samples analyzed.

DNA length	DNA copies per bead	DNA amount per bead (ng)	Measured DNA amount^b^ (ng)	Measured DNA concentration^b^ (fmol)
613 bp^a^	4.75 × 10^5^	3.15 × 10^–4^	0.1575	0.39
300 bp^a^	4.75 × 10^5^	1.54 × 10^–4^	0.077	0.39
300 bp	2.37 × 10^5^	7.69 × 10^–5^	0.03845	0.19
300 bp	4.75 × 10^4^	1.54 × 10^–5^	0.0077	0.039
300 bp	4.75 × 10^3^	1.54 × 10^–6^	0.00077	0.0039
300 bp	4.75 × 10^2^	1.54 × 10^–7^	0.000077	0.00039
300 bp	4.75 × 10^1^	1.54 × 10^–8^	0.0000077	0.000039
157 bp	9.08 × 10^5^	1.54 × 10^–4^	0.077	0.74
157 bp^a^	4.75 × 10^5^	8.05 × 10^–5^	0.04025	0.39
157 bp	4.75 × 10^4^	8.05 × 10^–6^	0.004025	0.039

^a^3 samples selected to study the effect of DNA length on impedance response (see Fig. 4).

^b^For each sample, measurements were performed using an aliquot of approximately 500 beads.

#### DNA quantification score

To quantify DNA fragment amounts and distinguish samples with different DNA concentrations, we developed a quantification score (Q-score), which is the summation of the IPR measured at all eight frequencies. For each sample, we averaged the IPR of each bead measured at the eight frequencies and then calculate the summation. The Q-score includes the difference in both lower and higher frequency, which gives an overview of differences in electrical properties of different DNA beads. It also helps to differentiate beads containing different DNA amounts, and in particular at low concentration levels, since using only a single frequency as beads may not be distinguishable at some frequencies. To account for device-to-device variation and day-to-day environmental variation (e.g., room temperature, humidity) which could potentially affect the impedance response, DNA sample beads were always analyzed in comparison to bare beads measured in the same experiment. This allowed for calibrating IPR of DNA coated beads with bare beads to account for possible variations. As the DNA amount on beads increased, the difference in Q-score between DNA beads and bare paramagnetic beads increased. We studied the linear correlation between quantification score and the logarithm of DNA quantity attached to the beads. Figure 2b shows the Q-score of beads with differing DNA concentrations compared to bare streptavidin coated paramagnetic beads. Results showed approximately two orders of magnitude dynamic range with a detection limit of 0.0039 fmol (quantity of DNA in 500 beads) for 300 bp DNA strands (p < 0.001), which was the equivalent to 1.54 fg per bead. The curve was flat below the detection limit because the DNA was no longer detectable. The high end of the linear dynamic range was 0.19 fmol as increased DNA sample concentration was not able to further increase the bead’s surface charge and IPR. A potential phenomenon for this is that binding capacity of the beads is affected by the high concentration of DNA. As mentioned in the brochure from Thermo Fisher on M280 beads, as more DNA strands are provided in the binding process, the binding capacity is affected by the steric availability and charge interaction between bead and DNA strands and between DNA strands. Thus, the IPR of DNA beads could not increase as the DNA concentration increases at high end. This seems also to be a phenomenon observed frequently in the context of various molecular assays^43–45^. Duplicate experiments were performed on the same device up to 4 times and using at least 500 beads per measurement to validate results.

#### Testing of 157 bp DNA beads

**Figure 3:**
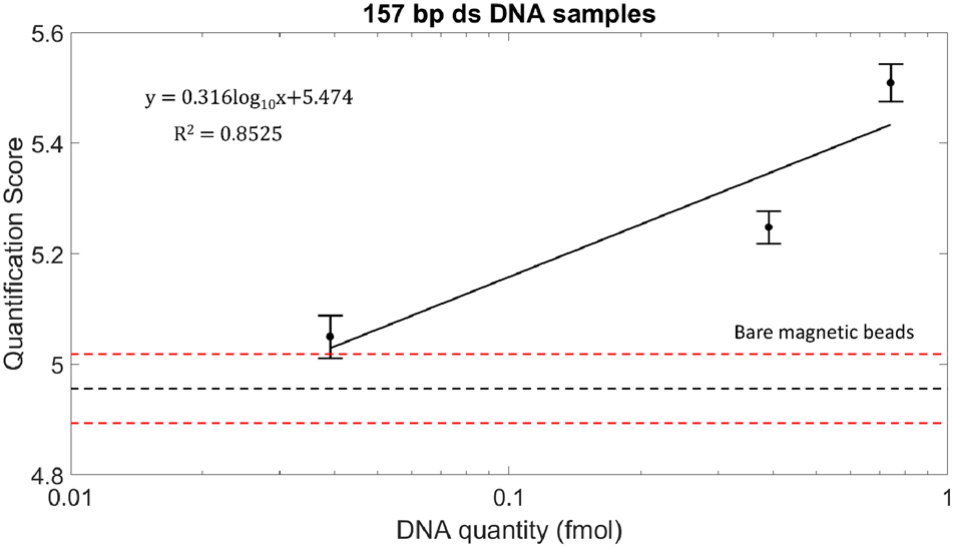
Effect of 157 bp ds DNA concentration on impedance response. The experiment on 157 bp ds DNA samples showed a similar trend with the impedance change directly related to increasing DNA amounts (Table 1). The impedance information from all 8 frequencies was converted into a quantification score used to plot the graph. Linear correlations between quantification score and the logarithm of 157 bp DNA fragments attached to the beads were calculated. Error bars represent standard error of mean. Horizontal black and red dotted lines represent the average quantification score of the negative control samples and negative control ± standard error, respectively.

We also measured DNA amounts for beads containing 157 bp double-stranded PCR products (Table 1). The IPR for these samples showed responses similar to those of the 300 bp DNA samples (Fig. S2) with a detection limit of 0.039 fmol. The Q-score plot is shown in Fig. 3. The R^2^ values indicated the linearity between two parameters (Q-score and nucleic acid quantity). The average Q-score of 0.039 fmol 157 bp DNA samples was at least one standard error above the upper bound (standard error) of the average Q-score of DNA-free control samples. These results demonstrated our device’s capability of quantifying DNA concentration based on multi-frequency impedance information. The results also illustrated that the detection limit of DNA concentration was length-sensitive. In addition to detecting PCR products of different lengths, we demonstrated the ability of our device for electronical detection of 30 base oligonucleotides with respective IPR responses and Q-score plots shown in SI Appendix (Fig. S3 and Fig. S4).

### Multi-frequency impedance response discriminates between different DNA sizes

**Figure 4:**
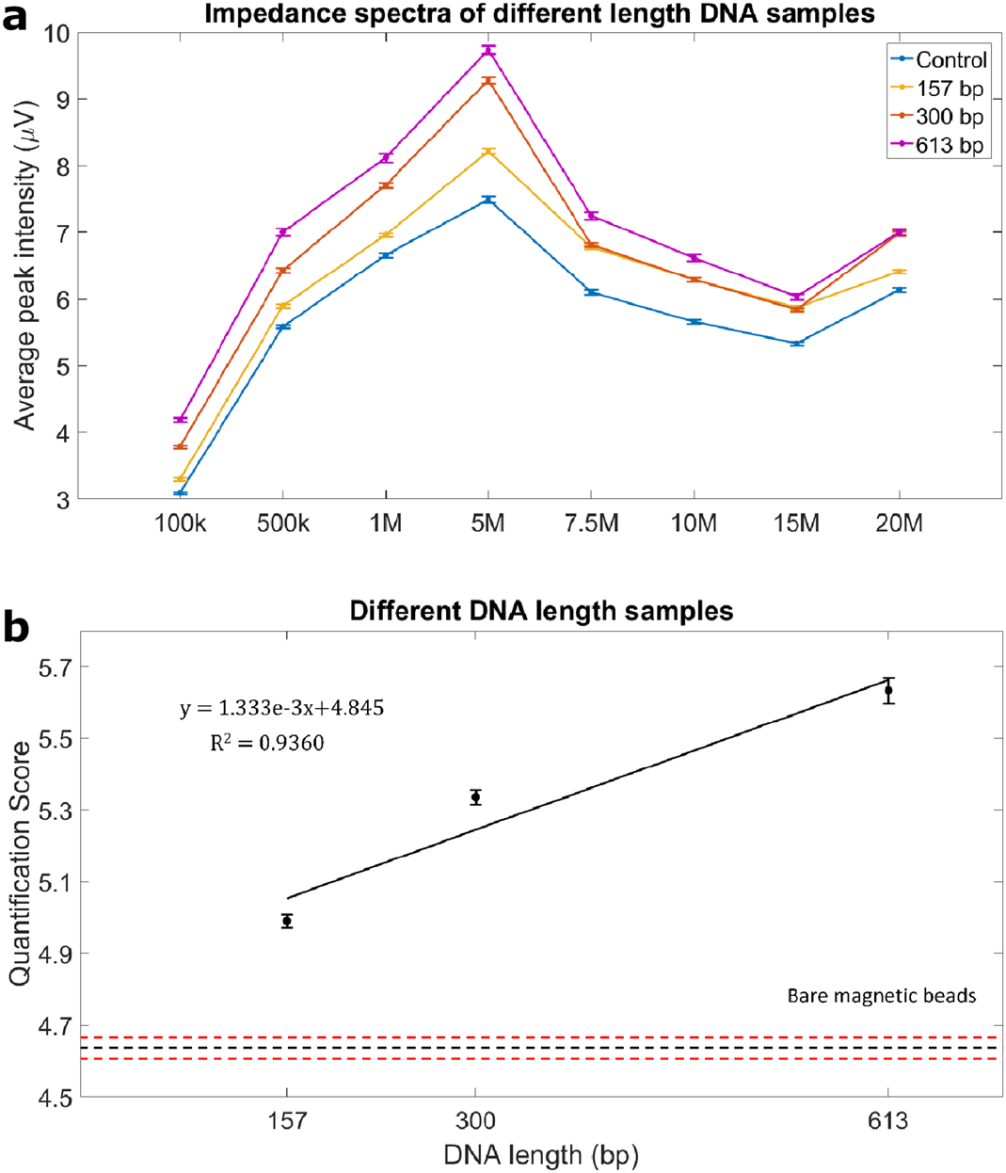
Effect of different DNA lengths of the same concentration on impedance response. Samples with different DNA lengths (ds 157 bp, ds 300 bp and ds 613 bp) but the same concentration (Table 1) were analyzed in comparison to bare paramagnetic beads (M280). (**a**) Impedance spectra for beads attached with different DNA length increased as frequency increased from 100 kHz to 5 MHz and then decreased as frequency increased to 15 MHz. (**b**) The quantification score of different length DNA beads showed a linear correlation between quantification score and DNA length attached to the beads. Error bars represent standard error of mean. Horizontal black and red dotted lines represent the average quantification score of the negative control samples and negative control ± standard error, respectively.

We investigated if the length of DNA fragments attached to beads could have an effect on electrical impedance. Average IPR was calculated by measuring the impedance of beads containing DNA fragments of different length but of the same molar concentration at eight different frequencies (Fig. 4a). The 613 bp DNA beads had the largest relative IPR for all frequencies tested, while beads coupled with 157 bp DNA had the smallest IPR. We also used the Q-score to identify the impedance difference between DNA beads and the negative control across all frequency measurements. As shown in Fig. 4b, the Q-score increased linearly with increasing DNA length. Since the number of copies per bead was identical for all samples, beads coated with longer DNA fragments showed a higher electrical surface potential and thus a higher average IPR. The average Q-score for 157 bp beads at 0.39 fmol was at least one standard error above the upper bound (standard error) of the average Q-score of DNA-free control beads.

## Discussion

In this work, we present a novel method for electronic detection and sizing of DNA fragments coupled onto paramagnetic beads using multi-frequency impedance cytometry. Our technology improves on previous electronic techniques that relied on particle diameter^40,46–49^ by capturing additional frequency-dependent properties such as conductivity and permittivity change. We show that DNA binding changes the surface conductivity of paramagnetic beads, which can be detected through frequency-dependent impedance peak response. IPR differed between DNA coated beads and bare streptavidin coated beads (negative control) as DNA concentration increased in the linear region of the dynamic range. We show the utility of this new technology for reliable detection of DNA fragments of different concentrations and lengths. This technology has potentially broader applications for detection and quantification of nucleic acid including mRNA and microRNA.

Starting from a genomic DNA sample, targeted PCR amplification of specific genomic regions is performed to reduce sample complexity and increase sensitivity and specificity for target detection. The coupling of DNA fragments to beads increases the relative impedance changes for DNA detection. The biotinylated DNA strands are easily captured onto streptavidin coated beads, and in combination with impedance flow cytometry, hundreds of beads can be continuously detected within minutes for rapid and highly sensitive DNA detection. This method requires only a small sample volume, which could be further reduced by technical optimization of the device. Given that DNA fragments are immobilized on beads instead of on the surface of the microfluidics channel, this technology is reusable and potentially portable. In future work, we envision utilizing barcoded beads, each carrying either a different length product or different target to perform multiplex analysis^50,51^. Measurements of the zeta potential of DNA bonded beads has been used to characterize the beads instead of quantifying DNA concentration in literatures^52–54^. However, it would be interesting to measure the zeta potential of DNA beads using our biosensor in future studies.

Currently, gel electrophoresis and real-time PCR are widely used in DNA quantification and sizing. Using optimized gel electrophoresis technology, Agilent Bioanalyzer can detect PCR products with a concentration as low as 0.1 ng within an analysis time of 30 min. In comparison, Real-Time PCR utilizes optical instrumentation and has a detection limit of several copies/μl or several fg/μl. While highly sensitive, real-time PCR has limitations in terms of DNA fragment size (e.g. amplicon size should be < 200 bp) and limited multiplexing capabilities^55,56^. Our impedance sensor in combination with microfluidics technology could potentially be designed for multiplex and portable applications, however, more work is needed to show technical and analytical feasibility for this approach. To further advance the technology towards clinical translation, several technical optimizations can be made to minimize device to device variations. These include incorporation of multiple electrodes for redundancy of measurements as well as temperature sensors to assess and correct for day-to-day variations. These improvements could result in further reduction of the number of beads required for accurate DNA detection, which could lower the limit of detection and further decrease analysis time. We found that 150 beads (Q-score of 4.32, p < 0.05) randomly selected from the approximately 500 beads measured per experiment was sufficient to distinguish beads containing DNA fragments from negative control beads. Another technical optimization could be the use of pulseless and noiseless pressure pumps that could help decrease the required sample volume and measurement time. We estimated the flow rate in the PDMS microfluidics channel at 0.05 μL/min by comparing to experiments performed using a syringe pump. Thus, less than 1 μL of sample was used for measurement in the experiment while the remaining 59 μL were used for maintaining the flow. These results indicate that measurements using this device could potentially be performed using much smaller DNA input amounts and in even shorter time. Importantly, clinical validation of this device will require establishing DNA standards with known concentration and length. Similar to gel-electrophoresis, these standards would be run together with every test sample (e.g., samples with unknown DNA length and concentration) taking advantage of the technique’s strength in making multiple relative measurements. Our experiments provide an approach to establish such standards and to quantify DNA fragments at high accuracy and precision at the femtomolar level and over a 100-fold dynamic range.

## Materials and methods

### Sample preparation

Target DNA was generated using biotinylated DNA oligonucleotides and Polymerase Chain Reaction (PCR). The purified DNA fragments were coupled to 2.8 μm bare paramagnetic beads via streptavidin–biotin linkage (Fig. 1c) using the procedure detailed as follows. Biotinylated oligos synthesized by IDT (Coralville, IA, USA) were used to amplify different fragment sizes (157 bp, 300 bp, 613 bp) of mitochondrial DNA under standard PCR conditions. The PCR product was purified using Qiaquick PCR purification kit (Qiagen) to remove any unincorporated biotinylated oligos. The PCR product was eluted in water, quantified and prepared for immobilization to the streptavidin coated dynabeads (M280) from Invitrogen (11205D). The dynabeads were washed as per the manufacturer’s recommendations. The purified biotinylated DNA was immobilized with the washed beads and incubated at room temperature for 15 min using gentle rotation at 2000 rpm. Different concentrations of DNA were bound to the paramagnetic beads (Table 1). The biotinylated DNA coated beads were then separated on a magnet for 2–3 min and subsequently washed 2–3 times as per manufacturer’s recommendations. Finally, the washed biotinylated DNA coated beads were resuspended in 10 µl of water, for final concentrations as listed in Table 1. A separate set of samples was generated for beads with similar DNA copy numbers for ds 157 bp, ds 300 bp and ds 613 bp fragments.

### Electrode and microfluidic channel fabrication

We used standard photolithography, electron beam evaporation, and lift-off processing to fabricate the sensing electrodes on a 3-in. glass wafer. The electrodes are 20 μm in width and the gap between two electrodes is 30 μm. The process started with spin coating a thin layer of positive photoresist (AZ5214, MicroChemicals GmbH) on the wafer. Followed by pre-bake, mask and wafer alignment, UV exposure, development and post-bake, the desired pattern was transferred from the mask to the wafer. We deposited a 5 nm adhesive layer of chromium and a 100 nm layer of gold sequentially using electron beam evaporation. Then, unwanted parts were lifted off by submerging the wafer in acetone. Microfluidic channel fabrication was divided into two steps: channel mold fabrication and channel fabrication. The channel mold was fabricated using soft lithography. A layer of SU-8 is patterned on a 3-inch silicon wafer involving spin coating, soft bake, exposure, post exposure bake, development, and finally hard bake. The size of channel is 30 μm in width and 15 μm in height. The channel pattern was transferred from the mold to a polydimethylsiloxane (PDMS) slab using the following process. Pre-polymer and curing agent were mixed at a ratio of 10:1. Then the mixture was poured onto the channel mold, degassed and baked at 80 °C for 30 min to allow for curing. Afterwards, the mold was peeled off and two holes (one 5 mm and one 1.2 mm) were punched through the PDMS to be used as inlet and outlet. The PDMS slab was covalently bonded onto the glass electrode chip by treating the two substrates with oxygen plasma.

### Multi-frequency impedance flow cytometry

Multi-frequency impedance cytometry was performed to measure the electrical impedance responses at 8 different frequencies, ranging from 100 kHz to 20 MHz. To enable capillary driven flow of the test sample, the microfluidic channel was made hydrophilic by treating the chip with oxygen plasma. Fluid flow was actuated by both capillary flow and gravity. The fluid height difference between the inlet and outlet causes a pressure gradient in the channel that drives the fluid. Subsequently, PBS was injected into the channel to preserve the hydrophilicity and improve impedance measurements. PBS was then withdrawn from the channel and beads to be tested were introduced from the inlet. Beads were diluted in 60 μL PBS to assure that only a single bead would pass the electrodes at any point in time and to maintain a continuous flow for more than 15 min without the use of a syringe pump, which could introduce interference and noise to electrical measurements. The impedance across the electrode pair was modulated by beads passing through due to perturbations of the electric field. The data was captured by a commercial multi-frequency lock-in amplifier (Zurich Instruments HF2A, Zurich, Switzerland). Eight different AC frequency signals generated by the same lock-in amplifier were superimposed to stimulate the electrode. The voltage was set at 0.4 V to maximize signal power while preventing electrode corrosion. The output voltage is proportional to the impedance between two electrodes. To minimize interference and noise from the environment, the device was placed in a grounded metal box (faraday cage) during measurements.

We tested beads containing different quantities and lengths of DNA. The impedance response data at each concentration/length of DNA were collected from approximately 500 beads. To account for device-to-device variation and perform data normalization, we measured the response of bare streptavidin coated paramagnetic beads in each set of experiments.

## Supplementary Information

Supplementary Information.

## Data Availability

The datasets generated during and/or analyzed during the current study are available from the corresponding author on reasonable request.
