# Supplementary material for: Multi-frequency impedance sensing for detection and sizing of DNA fragments
Source: Sci Rep. 2021 Mar 22;11:6490. doi: 10.1038/s41598-021-85755-9 (PMC7985362; doi:10.1038/s41598-021-85755-9)
Supplement: Supplementary file 1 — Supplementary Information. [file 41598_2021_85755_MOESM1_ESM.docx]

Supplementary Information for

Multi-frequency impedance sensing for detection and sizing of DNA fragments

Jianye Sui, Neeru Gandotra , Pengfei Xie, Zhongtian Lin, Curt Scharfe, Mehdi Javanmard

Curt Scharfe and Mehdi Javanmard

Email: [curt.scharfe@yale.edu](mailto:curt.scharfe@yale.edu) and [mehdi.javanmard@rutgers.edu](mailto:mehdi.javanmard@rutgers.edu)

**This PDF file includes:**

Supplementary text

Figs. S1 to S4

Table S1

Supplementary Information Text

**The effect of charge on impedance.** We investigated the effect of surface potential of beads on the frequency dependent impedance response using bare magnetic beads M-270 and M-280. M-270 beads have a more negative potential on the surface than M-280 beads. By implementing multi-frequency impedance cytometry, the impedance responses at different frequencies were collected. The experiment was repeated twice and similar results were observed. The impedance difference of two beads over 8 frequencies is reported in Fig.S1. The bead with larger surface potential (M-270) had a larger impedance than the other (M-280). The result indicated that the surface potential can affect the frequency dependent impedance response. More DNA strands attached to the bare magnetic beads can increase the surface potential, since the DNA has a negative charge due to the negatively charged phosphate component. Thus, we can take advantage of the impedance of bead-DNA conjugates at different frequencies to differentiate DNA quantity.


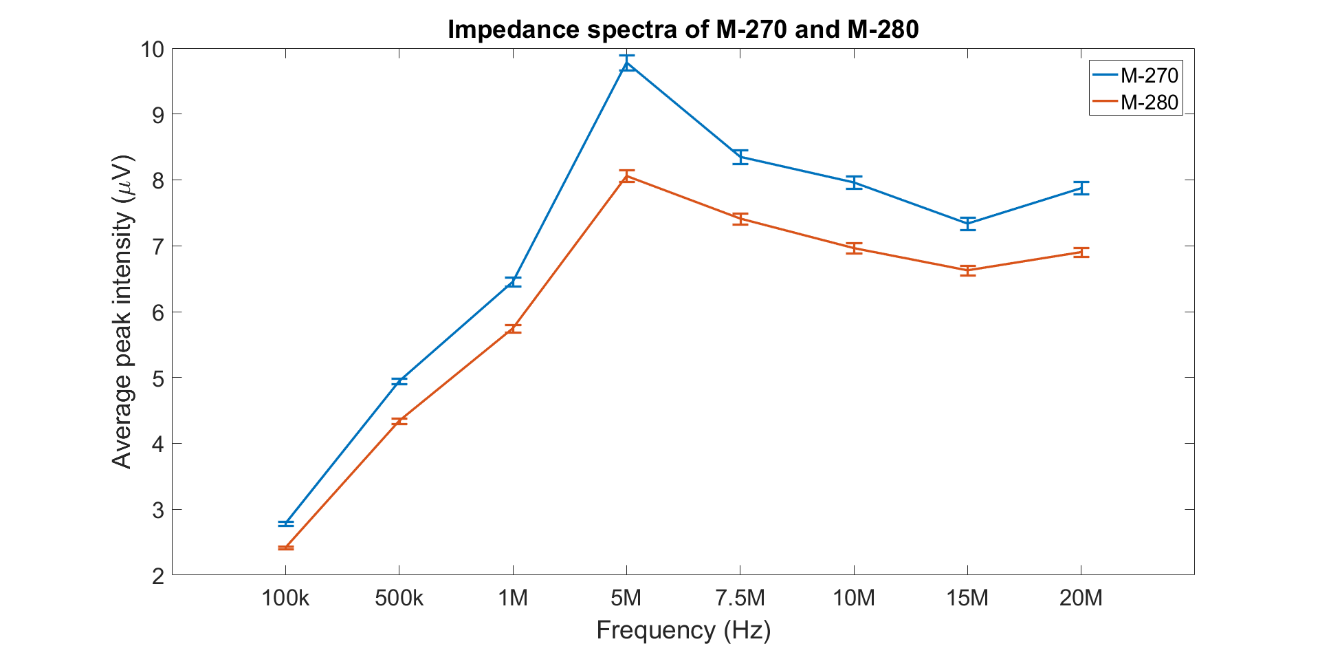


Fig. S1. Impedance spectra of beads with different surface potential (M-270 and M-280). At each single frequency, the average peak intensity of M-270 is larger than that of M-280.


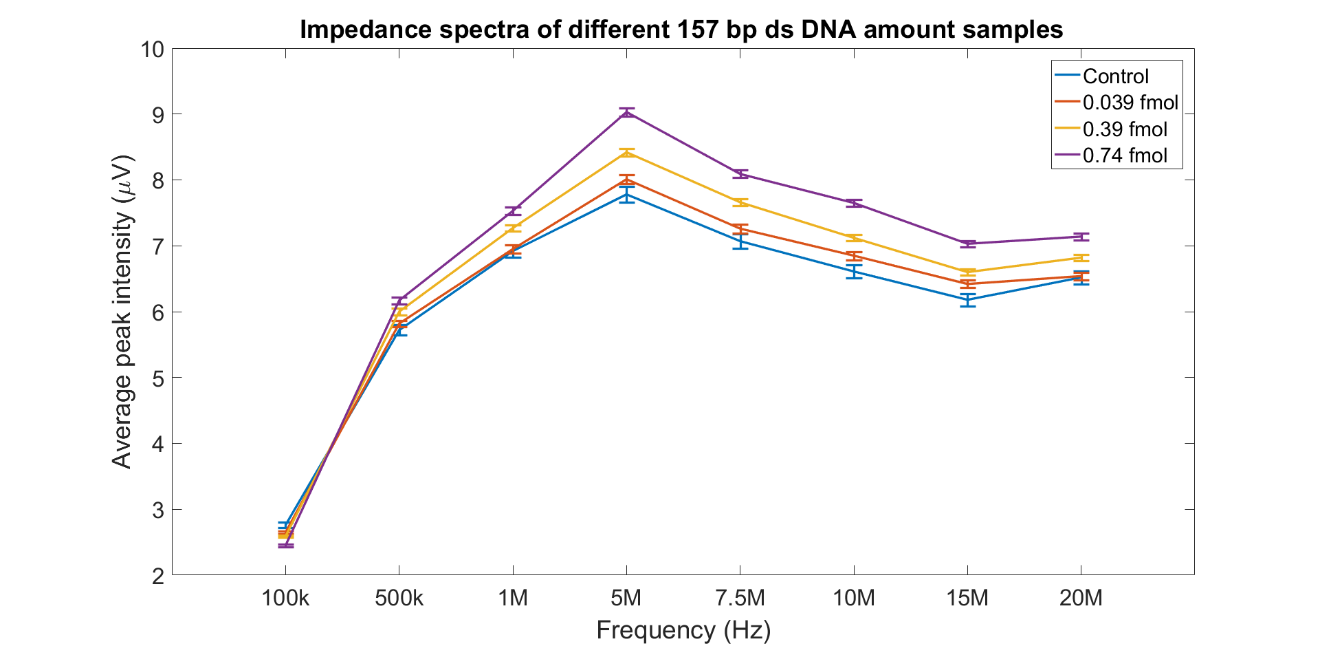


Fig. S2. Impedance spectra for beads attached with different 157 bp ds DNA amount (0.039 fmol, 0.39 fmol and 0.74 fmol) and negative control (bare magnetic M280 beads). The overall spectra is similar to 300bp samples results where peak intensity increases initially and then decreases. As the attached DNA amount increases, the average peak intensity increases, particularly from 5 MHz to 15 MHz.


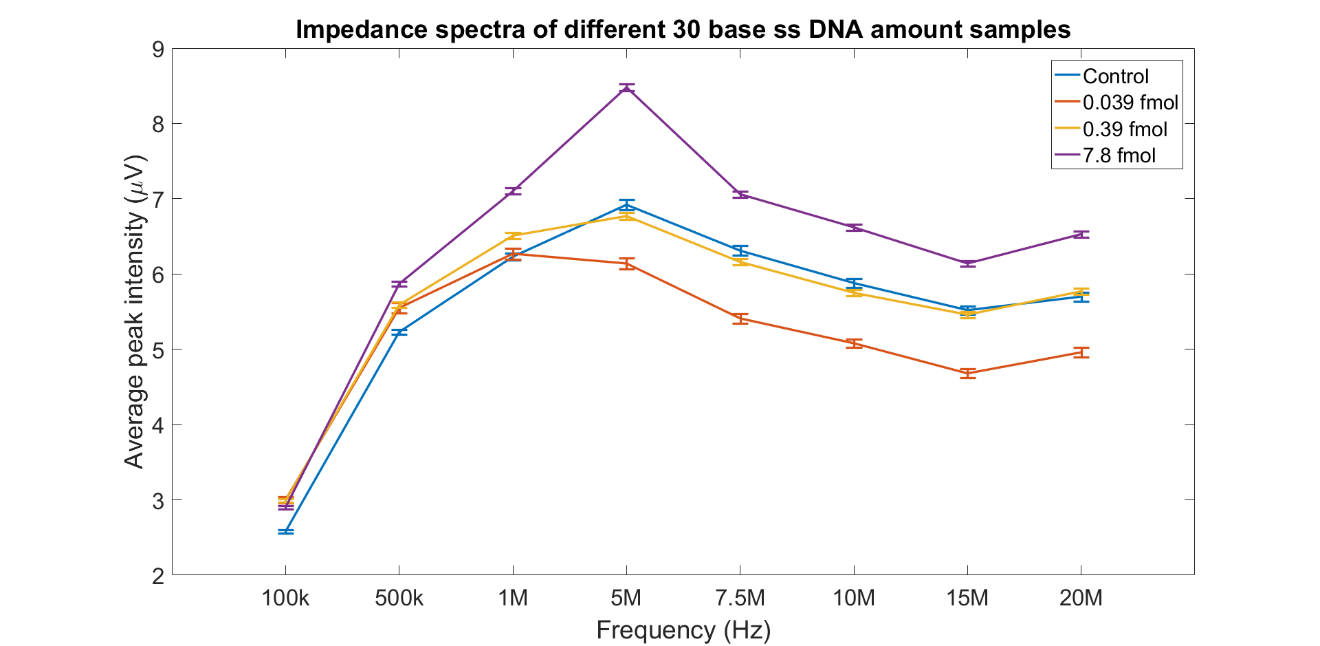


Fig. S3. Impedance spectra for beads attached with different 30 base ss DNA amount (Table. S1) and negative control (bare magnetic M280 beads). The 0.39 fmol DNA samples were not separable from bare magnetic beads, because of the detection limit.

**
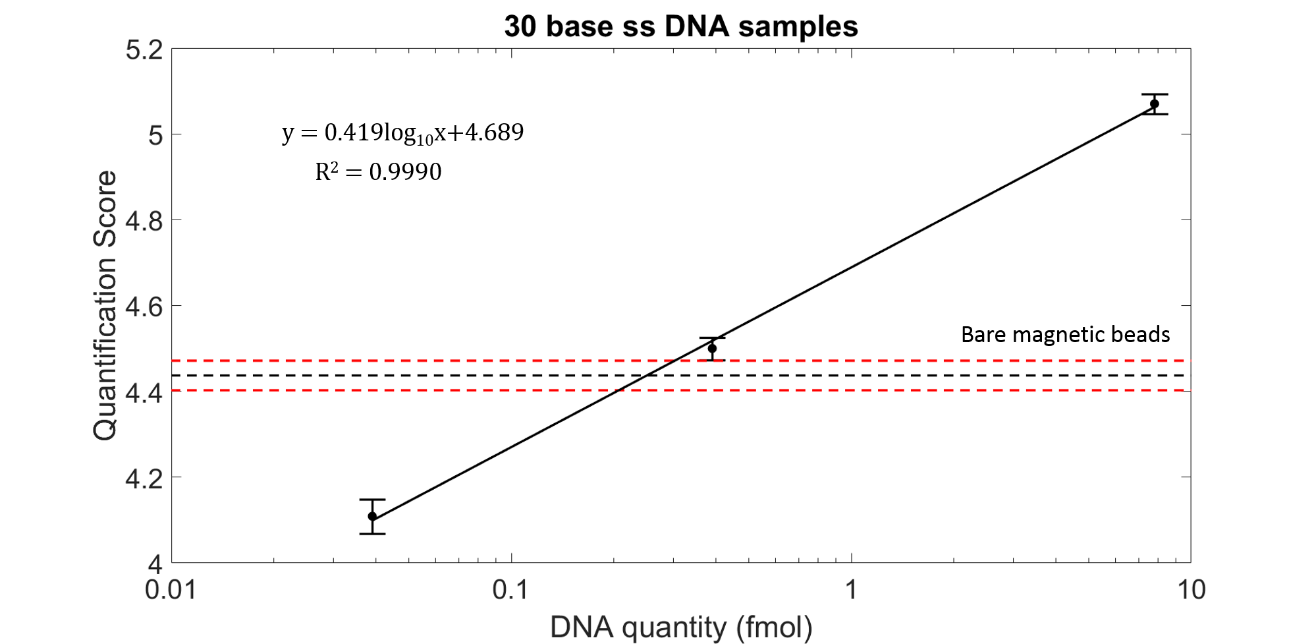
**

**Fig. S4.** Quantification score of beads with different 30 base ss DNA quantities (0.039 fmol, 0.39 fmol and 7.8 fmol) and negative control (bare paramagnetic M280 beads). Linear correlations between quantification score and the logarithm of 30 base single stranded DNA amount attached to the beads were calculated. Error bars represent standard error of mean. Horizontal black and red dotted lines represent the average quantification score of the negative control samples and negative control ± standard error, respectively.

Table. S1 Details of 30 base single stranded DNA beads

| DNA copies per bead | DNA amount per bead (ng) | Measured DNA amount^ (ng) | Measured DNA concentration^ (fmol) |
| --- | --- | --- | --- |
| 9.50×106 | 1.54×10-4 | 0.077 | 7.8 |
| 4.75×105 | 7.69×10-6 | 0.003845 | 0.39 |
| 4.75×104 | 7.69×10-7 | 0.0003845 | 0.039 |

^ For each sample, measurements were performed using an aliquot of approximately 500 beads.
